# Moderate obesity and endothelial dysfunction in humans: influence of gender and systemic inflammation

**DOI:** 10.1002/phy2.58

**Published:** 2013-08-28

**Authors:** Tisha Marie B Suboc, Kodlipet Dharmashankar, Jingli Wang, Rong Ying, Allison B Couillard, Michael J Tanner, Michael E Widlansky

**Affiliations:** 1 Department of Medicine, Division of Cardiovascular Medicine, Medical College of WisconsinMilwaukee, Wisconsin; 2 Department of Pharmacology, Medical College of WisconsinMilwaukee, Wisconsin; 3 Graduate School of Biomedical Sciences, Medical College of WisconsinMilwaukee, Wisconsin

**Keywords:** Adipose tissue, endothelial dysfunction, microvascular alterations, obesity, women

## Abstract

Our objective was to determine whether moderate obesity (Body Mass Index [BMI] ≥ 30 kg/m²) is associated with impaired conduit and microvascular endothelial function, and whether men or women are more susceptible to impairment of endothelial function related to moderate obesity. Forty-one middle aged, nondiabetic moderately obese (BMI 34.7 ± 4.0 kg/m^2^) and nonobese (BMI 24.3 ± 2.6 kg/m^2^) subjects of both sexes underwent noninvasive studies of endothelial function (brachial reactivity) and measurements of endothelial-dependent vasodilation of gluteal subcutaneous arterioles to acetylcholine (Ach). Endothelium-dependent vasodilation to Ach was decreased in the moderately obese compared with the nonobese (*P* < 0.001). Stratified analysis based on sex showed impairment of arteriolar endothelial function in women BMI ≥ 30 kg/m^2^ (*P* = 0.02), but not men. There was no difference between in vivo endothelial function flow-mediated dilation (FMD%) by BMI category. Sex-specific analysis showed FMD% was lower in women with BMI ≥ 30 kg/m^2^ compared to those with BMI < 30 kg/m^2^ (*P* = 0.02). No differences were seen in men based on BMI category (*P* = 0.18). In women, high sensitivity C-reactive protein (hsCRP) correlated with BMI (ρ = 0.68, *P* = 0.006). Moderate obesity is associated with impaired resistance arteriolar endothelial function. This is more prominent in women than men and is associated with systemic inflammation.

## Introduction

Obesity has well-described adverse effects on cardiovascular health (Poirier et al. 2006). Obesity-related changes to the arterial structure and function of medium and large arteries include increased arterial stiffness, noncompensatory dilatory remodeling, and impaired endothelial function, all of which are known to predict future adverse cardiovascular events (Ferreira et al. 2004a, 2004b; Grassi et al. 2009).

Resistance arteriolar remodeling and endothelial dysfunction, observed in patients with hypertension (HTN) and type 2 diabetes (T2DM), also predict the incidence of adverse cardiovascular events (Mulvany and Aalkjaer 1990; Vlachopoulos et al. 2003; Mathiassen et al. 2007; Levy et al. 2008). Obesity-related endothelial dysfunction is present in small resistance arterioles of morbidly obese patients (BMI ≥ 40 kg/m^2^) undergoing bariatric surgery (Grassi et al. 2009; De Ciuceis et al. 2011; Farb et al. 2012). Interestingly, endothelial dysfunction in this morbidly obese population is prevalent even in the absence of HTN and T2DM (Grassi et al. 2009).

Moderate obesity (BMI ≥ 30 kg/m^2^) is prevalent and the impact on arteriolar endothelial function in humans remains unknown. Furthermore, whether obesity impacts conduit and microvascular endothelial function in men and women in a similar fashion remains unknown. In this study, we evaluated nondiabetic subjects to determine the association of moderate obesity with impaired conduit and microvascular endothelial function. In addition, we also compared men and women to determine which is more susceptible to impairment of endothelial function in relation to moderate obesity.

## Methods

### Subjects

We reviewed subjects who had participated in published studies of endothelial function at the Medical College of Wisconsin between 2008 and 2012 (Dharmashankar et al. 2012; Kizhakekuttu et al. 2012). Study protocols were approved by the Medical College of Wisconsin's Institutional Research Board. Forty-one middle-aged, nondiabetic subjects (mean 49 ± 9 years) who underwent both brachial artery endothelial function testing (flow-mediated dilation, FMD) and evaluation of the microvascular endothelial function using acetylcholine (Ach) were identified. Study procedures were as previously described (Dharmashankar et al. 2012; Kizhakekuttu et al. 2012). Women were classified as postmenopausal by self-report of no menses for at least 1 full year prior to their study date.

### General procedures

Height and weight were measured. Waist circumference was measured at the level of the umbilicus while standing. Heart rate and blood pressure (BP) were measured in triplicate and averaged. Blood samples were drawn from a peripheral arm or forearm vein for plasma and serum biomarker analyses.

### Measures of in vivo endothelial function

#### Brachial artery reactivity testing

In vivo measurements of endothelial function were performed by FMD as previously described (Kizhakekuttu et al. 2010). Brachial artery images were captured using a high-resolution ultrasound. Images were recorded prior to and following analysis and BP cuff inflation. FMD measures the vasomotor response during reactive hyperemia phase, a widely used test of endothelial function (Poredos and Jezovnik 2012). We measured flow-mediated dilatation (FMD%), nitroglycerin-mediated dilatation (NMD%), flow velocity, and shear stress in the brachial artery as previously described (Kizhakekuttu et al. 2010). Due to the logistical complexities of scheduling these studies, not all premenopausal women could be studied during the same point in their menstrual cycle.

#### Measurement of in vitro endothelial function by videomicroscopy

Human adipose arterioles were obtained by gluteal adipose pad biopsy for evaluation of endothelial-dependent and independent vasodilation by videomicroscopy as previously described (Dharmashankar et al. 2012; Kizhakekuttu et al. 2012). A 1–1.5 cm incision was made into the cephalad, outer quadrant of the gluteal region on the patient's nondominant side to gain access to the subcutaneous fat. 2–2.5 mL of fat tissue was removed for dissection and isolation of arterioles. Our prior work demonstrates that endothelium-dependent vasodilation of these vessels to acetylcholine (Ach, Sigma, St. Louis) is ∼95% dependent on endothelium-derived nitric oxide (NO) synthase (Dharmashankar et al. 2012; Kizhakekuttu et al. 2012). Endothelium-dependent vasodilatation was evaluated by videomicroscopy using increasing concentrations of Ach from 10^−10^ to 10^−5^ mol/L. Vasodilation was recorded as percentage change from the baseline diameter following endothelin-1 preconstriction to 50–70% of the maximal diameter. Endothelium-independent vasodilation was determined using papaverine (Pap, 2 × 10^−4^ mol/L, Sigma-Aldrich, St. Louis, MO) at the end of each Ach dose–response curve.

### Statistical analysis

Statistical analyses were performed using SigmaStat 12.0 and SPSS 21.0. The baseline characteristics and in vivo endothelial function are expressed as mean ± SD and compared using unpaired-t or chi-square tests as appropriate. The Ach dose–response curves between subjects with BMI greater than or equal to 30 kg/m^2^ and BMI less than 30 kg/m^2^ were compared by 2-way mixed analyses of variance (ANOVA). In addition post hoc testing at each Ach dose was performed if *P* < 0.05 to evaluate an overall difference in the interaction between dose and percent vasodilation. We determined correlations between vasodilation to peak Ach and FMD% dose anthropomorphic characteristics, high sensitivity C-reactive protein (hsCRP), glucose, and insulin using Pearson's r or Spearman's ρ as appropriate based on variable distribution. These analyses were repeated with stratification by sex. *P-*values of <0.05 were considered statistically significant.

## Results

### Subject characteristics

Forty-one subjects were evaluated (21 women, 11 premenopausal, 10 postmenopause). The baseline characteristics for the entire cohort and the cohort stratified by sex are shown in Tables 1 and 2, respectively. The average BMI in the BMI < 30 kg/m^2^ group was 24.3 ± 2.6 kg/m^2^ versus 34.7 ± 4.0 kg/m^2^ for those with BMI ≥ 30 kg/m^2^ (*P* < 0.001). There were no significant differences in age, sex, history of HTN, smoking status, BP, lipid profiles, plasma insulin, average systolic (SBP), average diastolic blood pressure (DBP), and waist circumference. In addition to BMI, weight, fasting glucose, and C-reactive protein were significantly higher in the BMI ≥ 30 kg/m^2^ group. Subject medication histories were also recorded. One participant was on angiotensin converting enzyme inhibitor, three on hydrochlorothiazide, one on spironolactone, and one on estrogen. No participants were on beta-blockers, calcium channel blockers, or statins.

**Table 1:** Clinical characteristics – entire cohort

	Nonobese	Moderately obese	
	BMI < 30 (kg/m^2^)	BMI ≥ 30 (kg/m^2^)	
	Mean *N* = 21	Mean *N* = 20	*P*-value
Age	48.86 ± 8.70	46.3 ± 8.35	0.34
Male sex	62% (13)	38% (7)	0.12
History of hypertension	14% (3)	20% (4)	0.69
BMI (kg/m^2^)	24.3 ± 2.6	34.7 ± 4.0	<0.001*
Waist Circumference	87.42 ± 22.37	111.31 ± 32.70	<0.001*
SBP (mmHg)	120.33 ± 22.25	131.05 ± 16.35	0.08
DBP (mmHg)	73.63 ± 16.39	75.88 ± 10.12	0.60
LDL cholesterol (mg/dL)	99.33 ± 26.50	106.15 ± 19.86	0.35
HDL cholesterol (mg/dL)	60.1 ± 18.65	51.45 ± 14.64	0.10
Triglycerides	75.38 ± 28.15	88.65 ± 30.35	0.15
Total cholesterol (mg/dL)	174.43 ± 34.43	175.35 ± 23.27	0.92
Insulin	9.59 ± 3.40	16.35 ± 11.94 (11)	0.16
Fasting glucose	74.1 ± 12.69	85.4 ± 12.30	<0.01*
Insulin (μU/mL)	9.2 ± 3.1 (16)	9.2 ± 3.8 (13)	0.99
HOMA-IR	1.7 ± 0.7 (16)	2 ± 0.9 (13)	0.38
C-reactive protein	1.58 ± 1.47 (9)	5.05 ± 4.10 (11)	0.02*
Current smokers	29% (6)	20% (4)	0.71

All data are reported as mean ± SD.

* *P* < 0.05 for comparisons between moderately obese and nonobese subjects. Bold values represent significant differences in fasting glucose and C-reactive protein between moderately obese and nonobese subjects.

**Table 2:** Clinical characteristics by sex

	Men (*N* = 20)		Women (N = 21)	
		
	BMI < 30 kg/m^2^ (*N* = 13)	BMI ≥ 30 kg/m^2^ (*N* = 7)	BMI < 30 kg/m^2^ (*N* = 8)	BMI ≥ 30 kg/m^2^ (*N* = 13)
Age (years)	47 ± 7	44 ± 12	51 ± 11	47 ± 6
History of hypertension (# subjects)	3	1	0	3
Current smokers (# subjects)	4	1	2	3
BMI (kg/m^2^)	25.1 ± 2.3	**33.9** ± 2.9*	22.9 ± 2.7	35.1 ± 4.6*
Waist circumference (cm)	90.5 ± 7.7	112.0 ± 7.9*	82.4 ± 7.9	110.9 ± 10.1*
SBP (mmHg)	128 ± 23	125 ± 14	108 ± 13	134 ± 17*
DBP (mmHg)	80 ± 16	73 ± 6	62 ± 8	77 ± 2*
Total cholesterol (mg/dL)	180 ± 38	173 ± 29	166 ± 29	177 ± 21
LDL cholesterol (mg/dL)	101 ± 31	110 ± 21	97 ± 18	104 ± 19
HDL cholesterol (mg/dL)	64 ± 21	44 ± 12	54 ± 11	56 ± 15
Triglycerides (mg/dL)	73 ± 30	92 ± 25	78 ± 27	87 ± 34
Fasting glucose (mg/dL)	74 ± 12	92 ± 8	74 ± 14	81 ± 13
Insulin(μU/mL)	9.0 ± 3.3 (*N* = 9)	12.1 ± 3.2 (*N* = 4)	9.5 ± 3.0 (*N* = 7)	7.9 ± 3.5 (*N* = 9)
HOMA-IR	1.7 ± 0.7 (*N* = 9)	2.8 ± 0.8* (*N* = 4)	1.7 ± 0.7 (*N* = 7)	1.6 ± 0.6 (*N* = 9)
hs-CRP (mg/dL)	2.1 ± 1.3 (*N* = 8)	2.4 ± 0.4 (*N* = 4)	0.8 ± 0.8 (*N* = 7)	6.3 ± 4.3* (*N* = 8)

All data are reported as mean ± SD.

* *P* < 0.05 for the comparison within sex.

### Measurements of human arteriolar endothelial function in *ex vivo* subcutaneous arterioles

**Figure 1:**
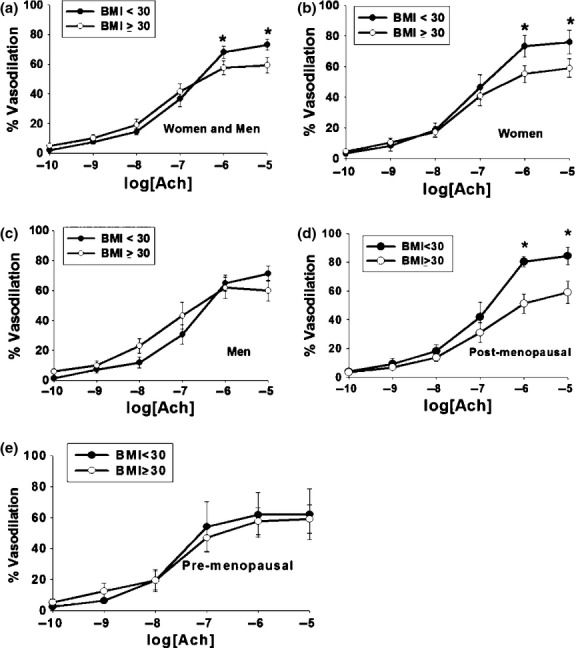
Ach Vasodilation in Human Gluteal Subcutaneous Adipose Arterioles. (A) Moderately obese (BMI ≥ 30 kg/m^2^) cohort had significantly decreased vasodilatory response of human gluteal subcutaneous arterioles during peak percent Ach-induced dilation (Ach 10^−5^ mol/L) compared to nonobese cohort (BMI < 30 kg/m^2^) (*P* < 0.001 overall, *P* < 0.05 at Ach 10^−6^ and 10^−5^ mol/L Ach concentrations) (*P < 0.05). (B) Sex-specific stratified analyses showed that resistance arteriolar endothelial function was impaired in women with BMI ≥ 30 kg/m^2^ (*P* = 0.02 overall, *P* < 0.05 at the 10^−5^ and 10^−6^ mol/L doses of Ach) (*P < 0.05). (C) Sex-specific stratified analyses demonstrated no significant resistance arteriolar endothelial function difference detected in men (*P* = NS). (D) Vasodilatory response of human gluteal subcutaneous arterioles during peak percent Ach-induced (Ach 10^−5^ mol/L) dilation response is significantly reduced in postmenopausal women with BMI ≥ 30 kg/m^2^ (*N* = 5 in each group, *P* = 0.03 overall, *P* < 0.05 at Ach doses 10^−6^ and 10^−5^ mol/L). (E) Vasodilatory response of human gluteal subcutaneous arterioles during peak percent Ach-induced (Ach 10^−5^ mol/L) dilation response showed no significant difference in premenopausal women with BMI ≥ 30 kg/m^2^ (*N* = 3 BMI < 30 kg/m^2^, *N* = 8 for BMI ≥ 30 kg/m^2^ *P* = 0.32 overall).

The arteriolar diameter (μm) did not differ between groups (95 ± 39 vs. 96 ± 34 μm for BMI < 30 kg/m^2^ and BMI ≥ 30 kg/m^2^, respectively, *P* = 0.99). Endothelium-dependent vasodilation to Ach was significantly blunted in individuals with BMI ≥ 30 kg/m^2^ (Fig. 1A). Endothelium-independent vasodilation to papaverine showed no significant difference between groups (98.8 ± 2.2% vs. 98.9 ± 3.2% BMI ≥ 30 kg/m^2^ compared to those with BMI < 30 kg/m^2^, *P* = 0.92). In addition, there were no sex-specific differences in vasodilation to papaverine (men: 99.0 ± 2.7%, women 98.7 ± 3.2% *P* = 0.74). As SBP trended higher in the obese group, we repeated these analyses adjusting for SBP. Despite adjustment for SBP, the significant impairment of endothelium-dependent vasodilation to Ach remained in the obese group (*P* < 0.001, data not shown). We performed stratified analyses based on sex and found resistance arteriolar endothelial function was impaired in women with BMI ≥ 30 kg/m^2^ compared to those with BMI < 30 kg/m^2^ (Fig. 1B). Adjustment for SBP and DBP did not affect the relationship between BMI ≥ 30 kg/m^2^ and impaired microvascular endothelial function in women (*P* = 0.005 overall). In men, while a significant difference was detected by the overall ANOVA (*P* = 0.02 for BMI group and Ach dose interaction), no significant differences were discerned by pairwise post hoc comparisons at each Ach dose (Fig. 1C).

We further subdivided the female study participants by menstrual status (pre- vs. postmenopausal). The difference in Ach response appeared primarily driven by postmenopausal women with BMI ≥ 30 kg/m^2^ (Fig. 1D). There was no significant difference in Ach-induced vasodilation by BMI in the premenopausal women (Fig. 1E).

### Measurements of in vivo endothelial function in obesity and males versus females

**Figure 2:**
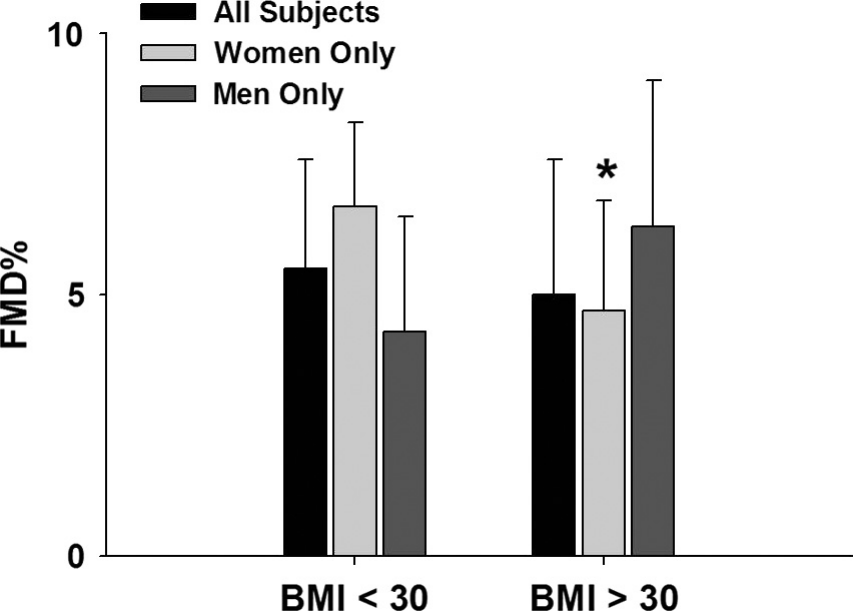
Overall and sex-specific results for brachial FMD% by BMI category. No differences in in vivo brachial artery endothelial function, as measured by FMD% (5.5 ± 2.1 vs. 5.0 ± 2.6% for BMI < 30 kg/m^2^ vs. BMI ≥ 30 kg/m^2^, respectively, *P* = 0.52). FMD% was significantly lower in women with BMI ≥ 30 kg/m^2^ compared to those with BMI < 30 kg/m^2^ (6.7 ± 1.6 vs. 4.3 ± 2.3% for BMI < 30 kg/m^2^ vs. BMI ≥ 30 kg/m^2^, respectively, *P* = 0.02).

Figure 2 shows the overall and sex-specific results for brachial FMD% by BMI category. Overall, we found no differences in in vivo brachial artery endothelial function, as measured by FMD% (). However, FMD% was significantly lower in women with BMI ≥ 30 kg/m^2^ compared to those with BMI < 30 kg/m^2^. When broken down by menstrual status, FMD% was significantly lower in postmenopausal women with BMI ≥ 30 kg/m^2^ (3.8 ± 1.7 vs. 6.4 ± 1.7% *P* = 0.04). There were no significant differences in FMD% for premenopausal women (*N* = 8 for BMI ≥ 30 kg/m^2^, *N* = 3 BMI < 30 kg/m^2^, 4.7 ± 2.6 vs. 7.2 ± 1.8% *P* = 0.16). No significant differences were seen in men based on BMI category (4.7 ± 2.1 vs. 6.3 ± 2.7%. for BMI < 30 kg/m^2^ vs. BMI ≥ 30 kg/m^2^, respectively, *P* = 0.18). While in women the baseline brachial diameter was significantly lower in the BMI < 30 kg/m^2^ group (*P* = 0.03), the correlation between diameter and FMD% in women was modest (*r* = −0.38, *P* = 0.09) and FMDmm did not significantly differ between BMI groups in women (0.21 ± 0.07 vs. 0.16 ± 0.08 mm for BMI < 30 and ≥30 groups, respectively, *P* = 0.16). There were no other overall or sex-specific significant differences in other brachial artery measures, including baseline and hyperemic shear stress, between BMI groups (Tables 3 and 4). Given the numerically greater number of male smokers in the BMI <30 group compared to BMI ≥ 30, we analyzed the data to determine if smoking status was confounding our FMD% data in men. We found no significant difference in FMD% (*P* = 0.95) for current male smokers (*N* = 5, FMD% = 5.2 ± 2.5) compared to nonsmokers (*N* = 15, FMD%=5.3 ± 2.5).

**Table 3:** In vivo measurements of endothelial function

Measurements	BMI < 30 (kg/m^2^) *N* = 21	BMI ≥ 30 (kg/m^2^) *N* = 20	*P*-value
Baseline diameter (mm)	3.76 ± 0.79	3.97 ± 0.54	0.34
Percent flow-mediated dilatation (FMD%)	5.50 ± 2.14	5.03 ± 2.56	0.52
Absolute flow-mediated dilation (FMDmm)	0.20 ± 0.10	0.20 ± 0.11	0.89
Resting shear (dynes/cm^2^)	38 ± 16	38 ± 18	0.98
Peak hyperemic shear (dynes/cm^2^)	77 ± 22	70 ± 29	0.38
Nitroglycerin-mediated dilation (NMD%)	23.70 ± 8.53 (14)	19.5 ± 5.47 (19)	0.09

All data are reported as mean ± SD.

**Table 4:** In vivo measurements of endothelial function by sex

	Men (*N* = 20)		Women (*N* = 21)	
		
	BMI < 30 kg/m^2^ (*N* = 13)	BMI ≥ 30 kg/m^2^ (*N* = 7)	BMI < 30 kg/m^2^ (*N* = 8)	BMI ≥ 30 kg/m^2^ (*N* = 13)
Baseline diameter (mm)	4.13 ± 0.62	4.30 ± 0.40	3.17 ± 0.68	3.79 ± 0.53*
Percent flow-mediated dilation (FMD%)	4.7 ± 2.1	6.3 ± 2.8	6.7 ± 1.6	4.3 ± 2.3**
Absolute flow-mediated dilation (FMD mm)	0.20 ± 0.11	0.27 ± 0.12	0.21 ± 0.07	0.16 ± 0.08
Resting shear (dynes/cm^2^)	31 ± 9	37 ± 13	50 ± 19	39 ± 21
Peak hyperemic shear (dynes/cm^2^)	67 ± 7	65 ± 21	101 ± 52	101 ± 65
Nitroglycerin-mediated dilation (NMD%)	23.6 ± 7.5 (*N* = 11)	18.4 ± 6.7 (*N* = 7)	24.0 ± 13.8 (*N* = 3)	20.2 ± 4.8 (*N* = 12)

All data are reported as mean ± SD.

* *P* = 0.03 versus Women with BMI < 30.

** *P* = 0.02 versus women with BMI < 30.

### Associations between microvascular and conduit vessel endothelial function and systemic modulators of endothelial function

**Figure 3:**
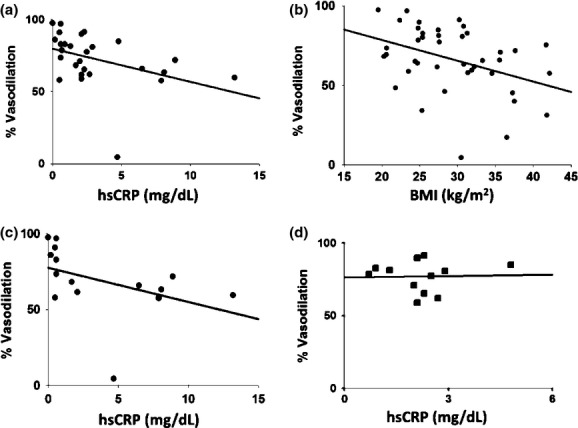
Correlations between Peak Percent Induced-Ach Dilation (Ach 10^−5^ mol/L) (A), hsCRP, and (B) BMI for the entire cohort and (C–D) sex-specific strata For the entire cohort, the only univariate predictors of the peak dose Ach-induced dilation include BMI (*r* = −0.39, *P* = 0.01) and hs-CRP (*r* = −0.51, *P* = 0.008, *N* = 27). HsCRP negatively correlated with peak Ach-induced vasodilation in women (ρ = −0.62, *P* = 0.02), however, hsCRP did not correlate with peak Ach vasodilation in men.

Analyses of univariate associations of peak Ach-induced vasodilation with other clinical variables for the entire cohort and in the sex-specific strata are shown in Table 5. For the entire cohort, the only univariate predictors of the peak dose Ach-induced dilation include BMI and hsCRP Age, waist circumference, lipid profile, fasting glucose, HOMA-IR (Homeostasis Model of Assessment-Insulin Resistance), QUICKI (quantitative insulin sensitivity check index), SBP, and DBP were not significant predictors of vasodilation to peak Ach dose. BMI strongly correlated with hsCRP (ρ = 0.64, *P* = 0.004). In men, age and BMI were borderline predictors of peak Ach-induced vasodilation. HsCRP did not correlate with peak Ach vasodilation in men, the correlation between hsCRP and BMI was attenuated (ρ = 0.34, *P* = 0.27), and no correlation was present between hsCRP and waist circumference (ρ = 0.49, *P* = 0.11). However, in women, hsCRP strongly correlated with BMI (ρ=0.68, *P* = 0.006) and had a borderline correlation with waist circumference (ρ=0.50, *P* = 0.06). hsCRP negatively correlated with peak Ach-induced vasodilation in women. Graphical depiction of the correlations between peak Ach, hsCRP, and BMI for the entire cohort and sex-specific strata are shown in Figure 3.

**Table 5:** Univariate association of peak ach-induced vasodilation response

	Overall (*N* = 41)		Men (*N* = 20)		Women (*N* = 21)	
			
	Correlation coefficient	*P*-value	Pearson's *r*	*P*-value	Pearson's *r*	*P*-value
Age	0.24	0.13	0.42	0.07	0.11	0.46
BMI	**−0.39**	0.01*	−0.40	0.08	−0.39	0.08
Waist Circumference	−0.26	0.10	−0.11	0.65	−0.29	0.21
SBP	−0.15	0.35	−0.32	0.17	−0.03	0.89
DBP	−0.19	0.24	−0.23	0.33	−0.19	0.42
Total Cholesterol	0.12	0.45	0.16	0.51	0.09	0.70
HDL	−0.10	0.45	−0.08	0.73	−0.14	0.56
LDL	0.22	0.16	0.25	0.30	0.22	0.34
Triglycerides	−0.004	0.98	0.04	0.86	−0.03	0.88
hs-CRP^1^	−**0.51**	0.008*	−0.02	0.97	**−0.61**	0.02*
Insulin (uU/ml)	0.16	0.42	−0.19	0.54	0.27	0.31
Glucose	−0.08	0.64	−0.02	0.94	−0.13	0.58
HOMA-IR^1^	0.11	0.56	−0.30	0.33	0.24	0.36

1 Correlation coefficient is Spearman's ρ rather than Pearson's *r* due to nonnormal distribution of the indicated variables.

* *P* < 0.05 for the comparison between sex and overall. Bold values represent significant association in hs-CRP and BMI with peak ach-induced vasodilation.

Brachial FMD% negatively correlated with hs-CRP (ρ = −0.47, P = 0.02) but not BMI (*r* = −0.14, *P* = 0.37) for the entire cohort. Similar to peak Ach-induced vasodilation, both BMI (*r* = −0.48, *P* = 0.03) and hsCRP (ρ = −0.68, *P* = 0.005) negatively correlated with FMD% in women. Neither BMI (*r* = 0.38, *P* = 0.13) nor hsCRP (ρ = −0.20, *P* = 0.53) correlated with FMD% in men. Full data on the univariate associations between FMD% with other clinical variables are included in Table 6.

**Table 6:** Univariate associations of FMD%

	Overall (*N* = 41)		Men (*N* = 20)		Women (*N* = 21)	
			
	Correlation coefficient	*P*-value	Correlation coefficient	*P*-value	Correlation coefficient	*P*-value
Age	0.12	0.47	0.16	0.52	0.08	0.73
BMI	−0.14	0.37	0.35	0.13	**−0.48**	0.03*
Waist Circumference	−0.09	0.56	0.01	0.97	**−0.55**	0.01*
SBP	−0.24	0.13	−0.15	0.54	−0.34	0.14
DBP	−0.24	0.13	−0.11	0.63	−0.39	0.08
Total Cholesterol	0.10	0.53	0.04	0.86	0.19	0.42
HDL	−0.002	0.99	−0.01	0.97	0.01	0.96
LDL	0.13	0.41	0.06	0.82	0.25	0.28
Triglycerides	−0.027	0.87	0.01	0.96	−0.06	0.79
hs-CRP^1^	−**0.47**	0.02*	0.20	0.53	−**0.68**	0.005*
Glucose	−0.03	0.86	0.09	0.70	−0.15	0.51
HOMA-IR^1^	−0.32	0.19	0.14	0.78	−0.46	0.14

1 Correlation coefficient is Spearman's ρ rather than Pearson's *r* due to nonnormal distribution of the indicated variables.

* *P* < 0.05 for the comparison between sex and overall. Bold values represent significant association in BMI, Waist Circumference, and hs-CRP with FMD%.

## Discussion

Our data demonstrate that moderate levels of obesity in nondiabetic humans impair resistance arteriolar endothelial function. We were unable to demonstrate a significant reduction in conduit vessel endothelial function in the overall group. However, we found that the relative impact of BMI ≥ 30 kg/m^2^ on both conduit and arteriolar endothelial function may differ by sex. Overall, our data reports three important novel findings. First, we extend prior work in morbidly obese humans (BMI ≥ 40 kg/m^2^) by demonstrating that endothelial dysfunction occurs in association with more modest and prevalent obesity levels (Arkin et al. 2008; Grassi et al. 2009; Farb et al. 2012). Furthermore, it suggests a novel moderate obesity sex-specific impact on endothelial function. Finally, we found endothelial dysfunction in obese women is also associated with inflammation.

To our knowledge, prior studies of the impact of obesity on resistance arteriolar endothelial function have exclusively investigated arterioles obtained from the abdominal subcutaneous or visceral adipose depots of morbidly obese patients (BMI ≥ 40 kg/m^2^) undergoing bariatric surgery (Arkin et al. 2008). Two studies in nondiabetic patients with insulin resistance report impairment in subcutaneous abdominal wall arteriolar endothelial function compared with lean control subjects (Grassi et al. 2009; De Ciuceis et al. 2011). A third study in a bariatric population with a 44% prevalence of diabetes found impaired visceral, but not subcutaneous, abdominal adipose arteriolar endothelial function. The reason for these conflicting findings is unclear. They may relate to differences in subject populations, in methods of measuring arteriolar endothelial function (micromyography vs. videomicroscopy), or potentially to differential effects of different anesthetics used during surgery in the different study settings. Due to overall small sample sizes in these previous studies and/or skewed enrollment toward one sex, there have been no prior studies evaluating sex-specific effect of obesity on arteriolar endothelial function. Our data importantly expands this knowledge by (1) extending the finding of obesity-associated endothelial dysfunction to the more accessible subcutaneous gluteal adipose depot where vessels can be obtained without the potential confounding use of general anesthesia, (2) demonstrating that the adverse impact of obesity on arteriolar endothelial function extends to a more modest and prevalent severity of obesity, and (3) suggesting that obesity, in the relative absence of other cardiovascular factors, may have greater impact on endothelial function in women relative to men.

In the human conduit circulation, FMD%'s inverse relationship with BMI and waist circumference has been demonstrated in multiple studies. (Hashimoto et al. 1998; Brook et al. 2001; Arkin et al. 2008) These data further suggest this relationship is more strongly related to visceral than subcutaneous adiposity (Brook et al. 2001; Marchesi et al. 2007; Parikh et al. 2009). Our disparate results in this study may be related to differences in study populations, size, and techniques of measuring endothelial function. Whether the relationships between measures of obesity (BMI and waist circumference) and FMD% differ by sex, where sex-related differences in fat distribution may impact cardiovascular risk, remains unclear (Donahue et al. 1987; Hartz et al. 1990; Ley et al. 1992). Emerging data call into question whether adiposity and FMD% are associated in men. While not excluding an association between BMI (or other measures of adiposity) and FMD% in men, our data suggest that inverse association between BMI and FMD% may be stronger in women. Further analyses of existing datasets and further studies are necessary to better evaluate the potential differential impact of obesity on vascular function based on sex.

Interestingly, we found BMI (a measure of overall adiposity) and hsCRP to be strongly correlated in women. HsCRP also had a strong, inverse correlation with both peak Ach dilation and FMD% in women. Over the past 2 decades, adipose tissue has emerged as a key pro-inflammatory organ whose inflammatory activity increases as adipose mass increases (Vincent and Taylor 2005). Adipose tissue produces systemically circulating interleukin-6, a known stimulant for the production of CRP (Vicennati et al. 2002; Vincent and Taylor 2005). Recent data, consistent with our findings, suggest women have higher levels of circulating CRP and that this is related to the greater accumulation of subcutaneous adiposity rather than visceral adiposity (Fichtlscherer et al. 2000; Ziccardi et al. 2002; Marchesi et al. 2007). Obese women who lose 10% of their weight in 1 year both improve their FMD% and have reduced their circulating levels of IL-6, tumor necrosis factor α (TNF-α), and soluble adhesion molecules (Ziccardi et al. 2002). Our findings extend these studies by demonstrating that, in women, arterioles from the subcutaneous gluteal adipose depot also express phenotypical obesity-related endothelial dysfunction related to systemic inflammation that may originate at least in part from their surrounding adipose.

We previously reported that endothelium-dependent vasodilation of the gluteal adipose arterioles is almost entirely dependent on endothelium-derived NO synthase (eNOS) activity (Dharmashankar et al. 2012). Our current findings suggest that obesity-associated arteriolar endothelial dysfunction in women is primarily related to impaired eNOS activity and/or NO bioavailability, similar to that seen in hypertensive and diabetic subcutaneous arterioles (Rizzoni et al. 2001; Widlansky et al. 2003; Tabit et al. 2010). Reduced adipose tissue blood flow present in obesity is mechanistically related to reduced NO bioavailability in the fasting state, and this reduced flow correlated with impaired FMD% in obese humans (Ardilouze et al. 2004; Funada et al. 2010). Our data confirm obesity-associated endothelial dysfunction in women is systemically present in both local and distant vascular beds and likely related to reduced NO bioavailability in both vascular beds.

In our study the waist circumference in women correlated with FMD% and not to peak Ach response. BMI correlated with both conduit and microvascular measures of endothelial function. The reasons behind these differences are unclear. Both subcutaneous and visceral adiposity are associated with impaired FMD% (Parikh et al. 2009). Gluteal subcutaneous arteriolar endothelial function may be more strongly associated to measures of overall adiposity like BMI. BMI includes both subcutaneous and visceral adiposity in contrast to waist circumference which is primarily a measure of visceral adiposity which is distant from the source of arterioles in this study. Regulation of subcutaneous arteriolar endothelial function likely relates in part to the arteriolar local subcutaneous environment which could account for the differences seen relative to BMI and waist circumference. Further study of the subcutaneous microenvironment relative to the endothelial phenotype of these microvessels is warranted.

Our data have several limitations. Our sample size of 41, while comparable to other key papers in this field, (Arkin et al. 2008; Grassi et al. 2009; Farb et al. 2012) is small. This suggests a need for independent validation by other groups to strengthen and support for our findings. We used BMI as a global measure of adiposity and waist circumference as a surrogate measure of visceral fat and did not specifically measure body composition or quantify subcutaneous and visceral fat. Also, we did not measure the structural differences between vessels by BMI category. The measure other systemic adipokines or local adipose inflammatory markers may also influence our phenotypes and merit further investigation. (Engeli et al. 2003; Shimabukuro et al. 2003; Arkin et al. 2008) While there were no significant associations between inflammation, BMI, waist circumference, and endothelial function in men in this study, we cannot conclude such associations do not exist owing to the small study sample. However, we can conclude that these associations are stronger in women. While our data suggest that postmenopausal versus premenopausal status could significantly influence the effects of obesity in women, these data should be considered hypothesis generating only given the small size of each subgroup and our inability to perform all studies in premenopausal women at the same time of their menstrual cycle which can affect FMD% measurements. Corroboration with future studies would help confirm this finding. Balanced against these limitations are the novelty of our findings extending the finding of obesity-related endothelial dysfunction to subcutaneous arterioles from individuals with more modest levels of obesity and demonstrating the potential for greater adverse impact of obesity on systemic inflammation and endothelial function in women relative to men.

Our data suggest that even moderate obesity (BMI ≥ 30), independent of diabetic status, adversely impacts microvascular and conduit endothelial function, particularly in women. This impairment appears most closely related to systemic inflammation which may originate in part from obesity-related excess adipose tissue. In light of the large body of data demonstrating endothelial dysfunction predicts adverse cardiovascular events, our data suggest moderate obesity, independent of its relationship to other risk factors, contributes to increased cardiovascular morbidity and emphasize the importance of weight control in both younger and older humans. Future work is necessary to better delineate the factors related to moderate obesity that lead to this impairment in both women and men.
